# Compressing atmospheric data into its real information content

**DOI:** 10.1038/s43588-021-00156-2

**Published:** 2021-11-25

**Authors:** Milan Klöwer, Miha Razinger, Juan J. Dominguez, Peter D. Düben, Tim N. Palmer

**Affiliations:** 1 Atmospheric, Oceanic and Planetary Physics, University of Oxford, Oxford, UK; 2 European Centre for Medium-Range Weather Forecasts, Reading, UK

**Keywords:** Computer science, Scientific data, Climate sciences, Information technology

## Abstract

Hundreds of petabytes are produced annually at weather and climate forecast centers worldwide. Compression is essential to reduce storage and to facilitate data sharing. Current techniques do not distinguish the real from the false information in data, leaving the level of meaningful precision unassessed. Here we define the bitwise real information content from information theory for the Copernicus Atmospheric Monitoring Service (CAMS). Most variables contain fewer than 7 bits of real information per value and are highly compressible due to spatio-temporal correlation. Rounding bits without real information to zero facilitates lossless compression algorithms and encodes the uncertainty within the data itself. All CAMS data are 17× compressed relative to 64-bit floats, while preserving 99% of real information. Combined with four-dimensional compression, factors beyond 60× are achieved. A data compression Turing test is proposed to optimize compressibility while minimizing information loss for the end use of weather and climate forecast data.

## Abstract (web summary)

Climate data are often stored at higher precision than is needed. The proposed compression automatically determines the precision from the data’s bitwise real information, removing any false information and leading to a more efficient compression.

## Main

Many supercomputing centers in the world perform operational weather and climate simulations several times per day^1^. The European Centre for Medium-Range Weather Forecasts (ECMWF) produces 230 TB of data on a typical day and most of the data are stored on magnetic tapes in its archive. This data production is predicted to quadruple within the next decade due to the increased spatial resolution of the forecast model^2–4^. Initiatives towards operational predictions with global storm-resolving simulations, such as Destination Earth^5^ or DYAMOND^6^, at a grid spacing of a couple of kilometers, will further increase the volume of data. These data describe physical and chemical variables for the atmosphere, ocean and land in up to six dimensions: three in space, as well as time, forecast lead time and the ensemble dimension. The last dimension results from calculating an ensemble of forecasts to estimate the uncertainty of predictions^7,8^. Most geophysical and geochemical variables are highly correlated in all of the dimensions, a property that is rarely exploited for climate data compression, although multidimensional compressors are being developed^9–12^.

Floating-point numbers are the standard to represent real numbers in binary form; 64-bit double-precision floating-point numbers (Float64) consist of a sign bit, 11 exponent bits representing a power of two, and 52 mantissa bits allowing for 16 decimal places of precision across more than 600 orders of magnitude^13^. Most weather and climate models are based on Float64 arithmetic, which has been questioned, as the transition to 32-bit single-precision floats (Float32) does not necessarily decrease the quality of forecasts^14,15^. Many bits in Float32 only contain a limited amount of information, as even 16-bit arithmetic has been shown to be sufficient for parts of weather and climate applications^16–19^. Shannon’s information theory^20,21^ introduced a mathematical concept to quantify information for the outcomes of a random variable. The information is analyzed in relation to the variable’s statistics or the statistical dependence on other variables and is often interpreted as the surprise about an outcome. Applied to binary numbers in simple chaotic dynamical systems, the information is zero for many of the 32 bits in Float32^22^. This supports the general concept of low-precision climate modeling for calculations and data storage, as, at least in theory, many rounding errors are entirely masked by other uncertainties in the chaotic climate system^23–25^.

The bitwise information content has been formulated for predictability in dynamical systems^22^. It quantifies how much individual bits in the floating-point representation contribute to the information necessary to predict the system’s state at a later point in time. This technique has been used to optimize the simulation of simple chaotic systems on inexact hardware to reduce the precision as much as possible. In this Article we extend the bitwise information content to distinguish between bits with real and false information in data. As false information leaves the result of data analyzes unaffected, only the real information is meaningful to analyze and should therefore be preserved in data compression.

Data compression for floating-point numbers often poses a trade-off in size, precision and speed^26–28^. Higher compression factors for smaller file sizes can be achieved with lossy compression, which reduces the precision and introduces rounding errors. Additionally, higher compression requires more sophisticated compression algorithms, which can decrease compression and/or decompression speeds. A reduction in precision is not necessarily a loss of real information, as rounding errors that occur are relative to a reference that itself comes with uncertainty. Here we calculate the bitwise real information content^20–22^ of atmospheric data to discard bits that contain no information^29,30^ and only compress the real information content. Combined with modern compression algorithms^10,31–33^, the multidimensional correlation of climate data is exploited for higher compression efficiency^34,35^.

## Results

### Drawbacks of current compression methods

The Copernicus Atmospheric Monitoring Service^36^ (CAMS) performs operational predictions with an extended version of the Integrated Forecasting System (IFS), the global atmospheric forecast model implemented by ECMWF. CAMS includes various atmospheric composition variables, such as aerosols, trace and greenhouse gases that are important to monitor global air quality. For example, the system monitors the spread of volcanic eruptions or emissions from wildfires. Most variables in CAMS have a multimodal statistical distribution, spanning many orders of magnitude (Supplementary Fig. 1).

The current compression technique used for CAMS is linear quantization, which is widely used in the weather and climate community through the data format GRIB2^37^. CAMS uses the 24-bit version, which encodes values in a data array with integers from 0 to 2^24^ − 1. These 24-bit unsigned integers represent values linearly distributed in the min–max range. Unused sign or exponent bits from the floating-point representation are therefore avoided, and some of the trailing mantissa bits are discarded in quantization. Choosing the number of bits for quantization determines the file size, but the precision follows implicitly, leaving the required precision or amount of preserved information unassessed.

Although linear quantization bounds the absolute error, its linear distribution is unsuited for most variables in CAMS: many of the available 24 bits are effectively unused as the distribution of the data and the quantized values match poorly (Supplementary Fig. 2). Alternately, placing the quantized values logarithmically in the min–max range better resolves the data distribution. As floating-point numbers are already approximately logarithmically distributed, this motivates compression directly within the floating-point format, which is also used for calculations in a weather or climate model and post-processing.

### Bitwise real information content

Many of the trailing mantissa bits in floating-point numbers occur independently and at similar probability, that is, with high information entropy^21,22^. These seemingly random bits are incompressible^38–40^, reducing the efficiency of compression algorithms. However, they probably also contain a vanishing amount of real information, which has to be analyzed to identify bits with and without real information. The former should be conserved while the latter should be discarded to increase the compression efficiency.

**Fig. 1:**
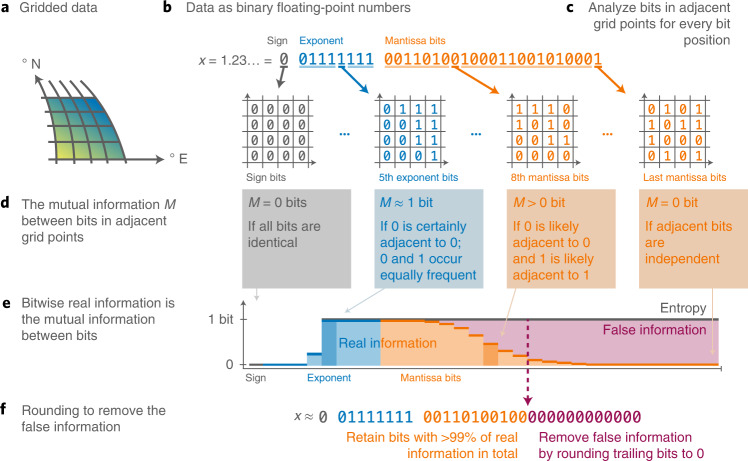
The bitwise real information content is defined as the mutual information of bits in adjacent grid points. **a**, Gridded data from a Cartesian, curvilinear or unstructured grid. **b**, Binary representation of every number in the data array. **c**, Analysis of the bits in the same bit position but from adjacent grid points. **d**, Calculation, for every bit position, of the mutual information between adjacent grid points, which is the bitwise real information content. **e**, A bit position contains more real information the stronger the statistical dependence to the adjacent bits is. Statistically independent bits contain only false information, which equals the entropy minus the real information. **f**, Bit positions that contain more than 99% of real information are preserved, while false information bits are removed by rounding to 0 to facilitate lossless compression.

We define the bitwise real information content as the mutual information^20,38,41–44^ of bits in adjacent grid points (Fig. 1 and Methods). A bit contains more real information the stronger the statistical dependence to the adjacent bits is. Bits without real information are identified when this dependence is insignificantly different from zero and we regard the remaining entropy in these bits as false information. The adjacent bit can be found in any of the dimensions of the data, for example, in longitude, time or in the ensemble dimension. However, the same bit position is always analyzed, for example, the dependence of the first mantissa bit with other first mantissa bits in adjacent grid points.

In general, this analysis can be applied to any *n*-dimensional gridded data array when its adjacent elements are also adjacent in physical space, including structured and unstructured grids. However, data without spatial or temporal correlation at the provided resolution will be largely identified as false information due to the independence of adjacent grid points (Supplementary Figs. 3 and 4 and Methods). If valuable scientific information is present in such seemingly random data, then the bitwise real information content as defined here is unsuited.

Jeffress et al. formulate the bitwise information content for simple chaotic systems, assuming an inherent natural uncertainty that had to be defined^22^. Their approach aims to enable reduced precision simulations on inexact hardware. Here we reformulate the bitwise real information as the mutual information in adjacent grid points for application in climate data compression. The quantization in the floating-point representation is used as an uncertainty, such that no additional assumption on the uncertainty of the underlying data has to be made. Most data compression techniques leave the choice of the retained precision to the user. The analysis here automatically determines a precision from the data itself, based on the separation of real and false information bits.

**Fig. 2:**
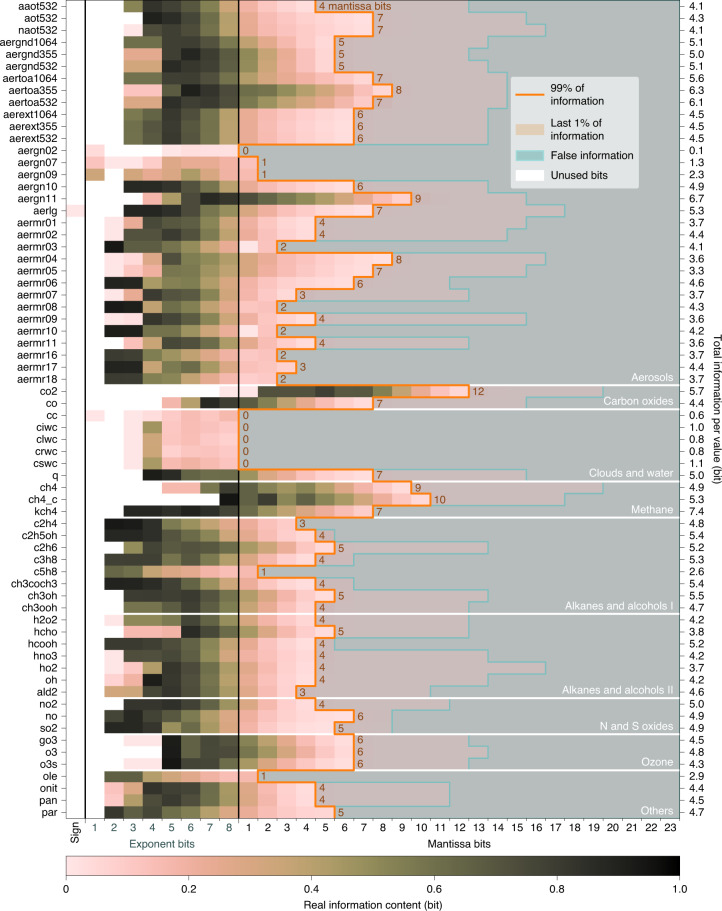
Bitwise real information content for all variables in CAMS. For each variable, the bitwise real information content is calculated in all three spatial dimensions in the 32-bit encoding of single-precision floats, revealing false information and unused bits. The bitwise real information content (gradient scale) is explained in Fig. 1. The mantissa bits that contain at least 99% of real information are enclosed in orange. Bits without any real information are shaded in gray-blue. The sum of the real information across bit positions per variable is the total information per value. Variable abbreviations are explained in Supplementary Table 1. Source data

Many exponent bits of the variables in CAMS have a high information content (Fig. 2), but the information content decreases within the first five to ten mantissa bits for most variables, such that many trailing mantissa bits do not contain real information. Exceptions occur for variables like carbon dioxide (CO_2_) with mixing ratios varying in a very limited range of 0.5–1.5 mg kg^−1^ (equivalent to ~330–990 ppmv) globally. Because of the limited range, most exponent bits are unused and the majority of the real information is in mantissa bits 2 to 12.

The sum of real information across all bit positions is the total information per value, which is less than 7 bits for most variables. Importantly, the last few percent of total information is often distributed across many mantissa bits. This presents a trade-off with which, for a small tolerance in information loss, many mantissa bits can be discarded, resulting in a large increase in compressibility (Supplementary Fig. 5). Aiming for 99% preserved information is found to be a reasonable compromise.

### Compressing only the real information

Based on the bitwise real information content, we suggest a strategy for the data compression of climate variables. First, we diagnose the real information for each bit position. Afterwards, we round bits with no significant real information to zero, before applying lossless data compression. This allows us to minimize information loss but maximize the efficiency of the compression algorithms.

Bits with no or only little real information (but high entropy) are discarded via binary round-to-nearest as defined in the IEEE-754 standard^13^ (Methods). This rounding mode is bias-free and therefore will ensure global conservation of the quantities that are important in climate model data. Rounding removes the incompressible false information and therefore increases compressibility. Although rounding is irreversible for the bits with false information, the bits with real information remain unchanged and are bitwise reproducible after decompression. Both the real information analysis and the rounding mode are deterministic, also satisfying reproducibility.

Lossless compression algorithms can be applied efficiently to rounded floating-point arrays (the round + lossless method). Many general-purpose lossless compression algorithms are available^39,40,45–50^ and are based on dictionaries and other statistical techniques to remove redundancies. Most algorithms operate on bitstreams and exploit the correlation of data in a single dimension only, so we describe such methods as one-dimensional (1D) compression. Here, we use the Zstandard algorithm for lossless compression, which has emerged as a widely available default in recent years (Methods).

**Fig. 3:**
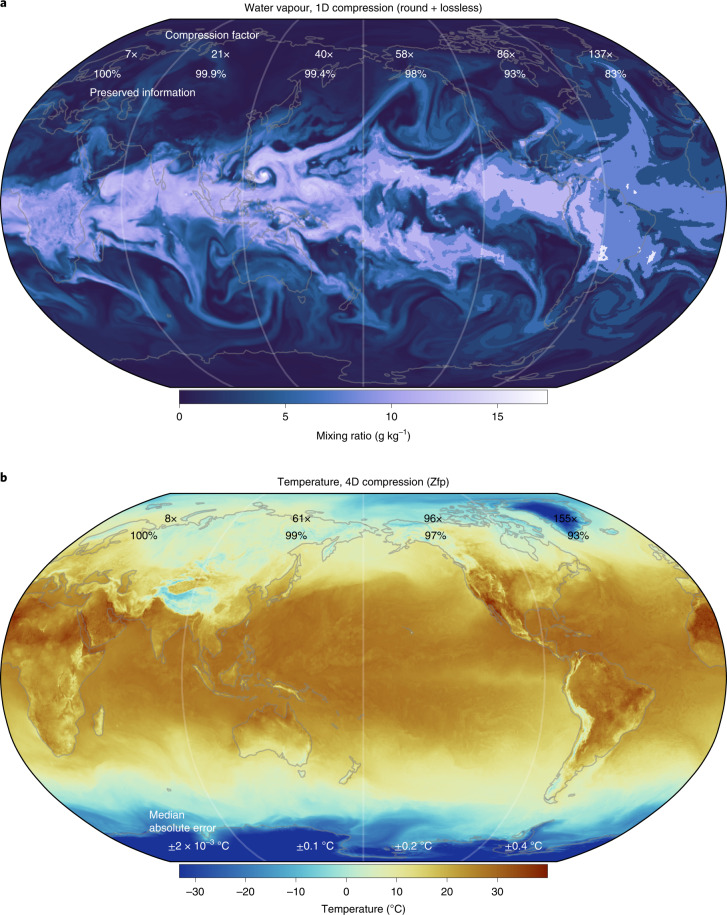
Compression at various levels of preserved information. **a**, Water vapor (specific humidity) compressed in the longitudinal dimension. **b**, Temperature compressed in the four space–time dimensions with compression algorithm Zfp. Preserved information decreases from left to right, which increases the compression factors relative to 64-bit floats. The vertical level shown is at ~2 km geopotential altitude in **a** and surface in **b**, but the compression factors include all vertical levels. Source data

The compression of water vapor at 100% preserved information (16 mantissa bits are retained) yields a compression factor of 7× relative to 64-bit floats (Fig. 3a). At 99% preserved information (seven mantissa bits are retained) the compression factor increases to 39×. As the last 1% of real information in water vapor is distributed across nine mantissa bits, we recommend this compromise to increase compressibility. With this compression a 15-fold storage efficiency increase is achieved compared to the current method (at 2.67×). Effectively only 1.6 bits are therefore stored per value.

**Fig. 4:**
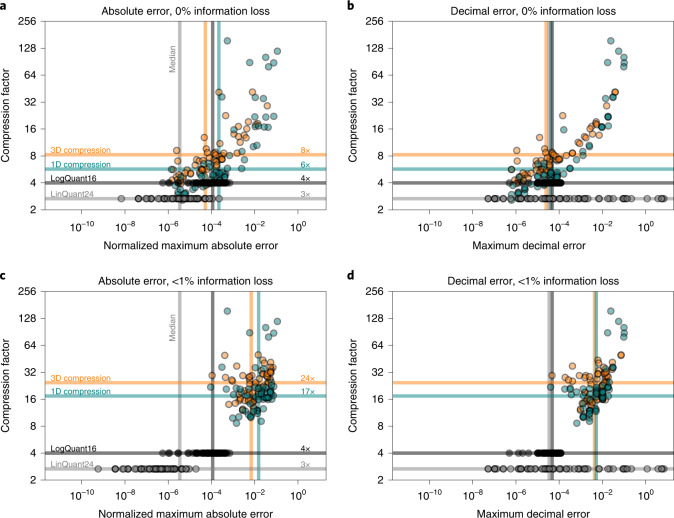
Compression factors versus compression errors. **a**–**d**, The maximum absolute and decimal errors for 24-bit linear and 16-bit logarithmic quantization (LinQuant24, LogQuant16) with 1D round + lossless and 3D Zfp compression are shown. Every marker represents, for one variable, the global maximum of the normalized absolute error (**a**,**c**) and decimal error (**b**,**d**) for 100% preserved information (**a**,**b**) and 99% preserved information (**c**,**d**). The geometric means of compression factors over all 68 variables are provided as horizontal lines. The medians of the errors across all 68 variables are given as vertical lines. Source data

Compressing all variables in CAMS and comparing error norms reveals the advantages of the 1D round + lossless method compared to the 24-bit linear quantization technique currently in use (Fig. 4). Owing to the logarithmic distribution of floating-point numbers, the round + lossless method has smaller maximum decimal errors (Methods and equation (15)) than the linear quantization for many variables. Some variables are very compressible (>60×) due to there being many zeros in the data—this is automatically made use of in the lossless compression. Compression factors are between 3× and 60× for most variables, with a geometric mean of 6× when preserving 100% of information. On accepting a 1% information loss, the geometric mean reaches 17×, which is the overall compression factor for the entire CAMS dataset achieved with this method. Furthermore, the 24-bit linear quantization could be replaced by a 16-bit logarithmic quantization, as the mean and absolute errors are comparable. The decimal errors are often even lower and naturally bound in a logarithmic quantization, despite there being fewer available bits.

The broad applicability of the bitwise real information content analysis for compression was tested with further datasets. Radar-based observations of precipitation over Great Britain are similarly compressible using the same method (Supplementary Fig. 6), as are satellite measurements of brightness temperature, with a very high resolution of ~300 m horizontally (Supplementary Fig. 7). Even for anthropogenic emissions of methane or nitrogen dioxide, similar compression results are obtained, despite the limited spatial correlation of the point sources (Supplementary Fig. 8). The bitwise real information content in this case is largely determined by the smooth background concentrations and is therefore still sufficiently high to preserve the point sources.

In an operational setting we recommend the following workflow. First, for each variable, the bitwise real information content is analyzed from a representative subset of the data. For example, a single time step can be representative of subsequent time steps if the statistics of the data distribution are not expected to change. From the bitwise real information, the number of mantissa bits to preserve 99% of information is determined (the ‘keepbits’). Second, during the simulation, the arrays that will be archived are rounded to the number of keepbits (which are held fixed) and compressed. The first step should be done offline—once in advance of a data-producing simulation. Only the second step has to be performed online, meaning every time a chunk of data is archived.

The presented round + lossless compression technique separates the lossy removal of false information and the actual lossless compression. This provides additional flexibilities, as any lossless compressor can be used, and application-specific choices can be made regarding availability, speed and the resulting file sizes. However, most general-purpose lossless compression algorithms operate on bitstreams and require multidimensional data to be unraveled into a single dimension. Multidimensional correlation is therefore not fully exploited in this approach.

We extend the ideas of information-preserving compression to modern multidimensional compressors. Analysis of the bitwise real information content leads naturally to the removal of false information via rounding in the round + lossless method. For other lossy compressors, however, the separation of real and false information has to be translated to the precision options of such compressors. Although such a translation is challenging in general, in the next section we present results from combining the bitwise real information analysis with one modern multidimensional compressor.

### Multidimensional data compression

Modern compressors have been developed for multidimensional floating-point arrays^10,31,32^ that compress in several dimensions simultaneously. We will compare the 1D round + lossless compression to Zfp, a modern compression algorithm for two to four dimensions^10^. Zfp divides a *d*-dimensional array into blocks of 4^*d*^ values (that is, an edge length of 4), which allows us to exploit the correlation of climate data in up to four dimensions. To extend the concept of information-preserving compression to modern compressors like Zfp, the bitwise real information is translated to the precision options of Zfp (more details are provided in the Methods).

Multidimensional compression imposes additional inflexibilities for data retrieval: data are compressed and decompressed in larger chunks, which can increase the load on the data archive. For example, if the data are compressed in time, several time steps have to be downloaded and decompressed, although only a single time step might be requested. Downloads from an archive might therefore increase if the data chunking is not well suited to typical data requests from users.

**Fig. 5:**
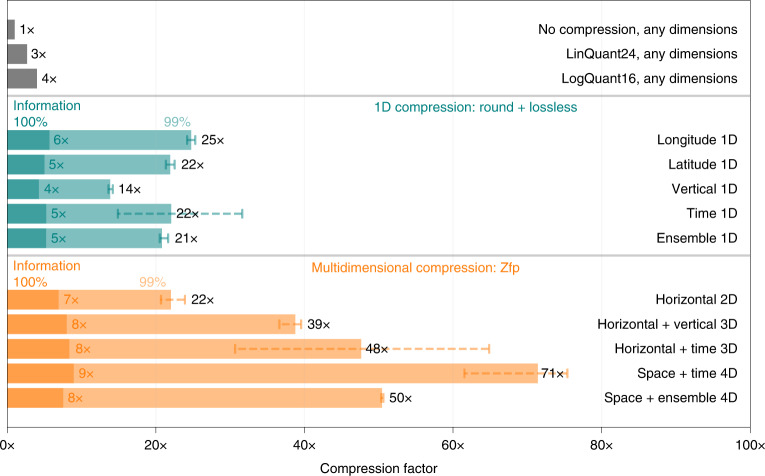
Compressing temperature’s real information in different dimensions. One-dimensional compression (round + lossless) with 99% and 100% preserved information in various dimensions is compared with 2D, 3D and 4D compression using Zfp. Error bars represent the minimum–maximum range of compression when repeated for the 91 vertical levels, for the 25 ensemble members and 125 time steps. Source data

For 1D compression, the compressibility varies with the dimension. Longitude (that is, in the zonal direction) is more compressible (reaching 25× for temperature at 99% preserved information) than the vertical (which yields only 14×) (Fig. 5). This agrees with the predominantly zonal flow of the atmosphere as spatial correlation in the zonal direction is usually highest. For a constant number of retained mantissa bits, higher resolution in the respective dimensions increases the compressibility as the correlation in adjacent grid points also increases (Supplementary Figs. 3 and 4).

For multidimensional compression it is generally advantageous to include as many highly correlated dimensions as possible. In that sense, including the hourly-resolved forecast lead time instead of the vertical dimension in 3D compression yields higher compression factors. The 4D space–time compression is the most efficient, reaching 60–75× at 99% preserved information. For temperature, this is equivalent to a median absolute error of 0.1 °C (Fig. 3b).

Compressing the entire CAMS dataset in the three spatial dimensions with Zfp while preserving 99% of the information yields an overall compression factor of 24× (Fig. 4). Maximum absolute error and decimal errors are, for most variables, very similar to 1D round + lossless compression (see Methods for a discussion of why they are not identical). This provides evidence that a multidimensional compression is preferable for higher compression factors.

**Fig. 6:**
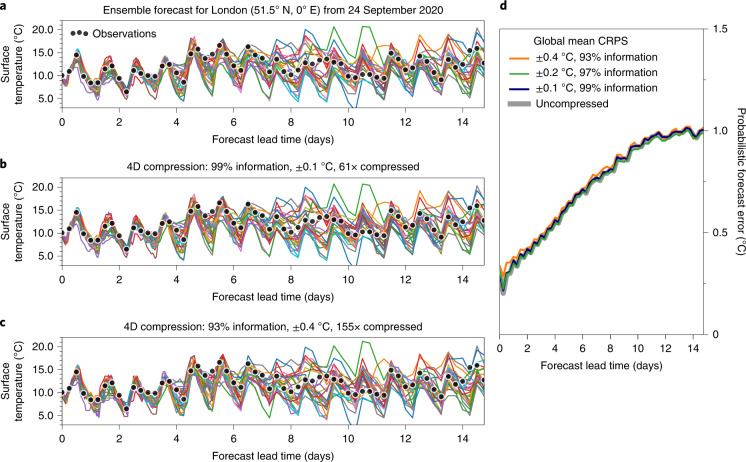
Verification of an ensemble forecast with the probabilistic forecast error based on ensemble data with and without compression. **a**, The 25-member uncompressed ensemble forecast (lines) of surface temperature in London, UK from 24 September 2020 up to 15 days ahead. **b**, Same as for **a** but the data are compressed in 4D space–time with Zfp, preserving 99% of real information. **c**, Same as for **b** but only preserving 93% of real information. **d**, Probabilistic forecast error (CRPS) for various levels of preserved information in the compression. The global mean CRPS has been calculated from 1,800 × 901 grid points. Source data

The meaning of error norms is limited in the presence of uncertainties in the uncompressed reference data. We therefore assess the forecast error to quantify the quality of the compressed atmospheric data. The continuous ranked probability score^51–53^ (CRPS) was evaluated for global surface temperature using observations every 6 h as truth (Fig. 6). The CRPS is the root-mean-square error between the observations and the forecast, but generalized to an ensemble of forecasts, accounting for both the ensemble spread and the bias. Compared to the uncompressed data, no significant increase in the CRPS forecast error occurs for individual locations or globally at 99% and 97% preserved information. The usefulness for the end user of the global temperature forecast is therefore unaltered at these levels of preserved information in the compression. However, with an information loss larger than 5%, the CRPS forecast error starts to increase, although large compression factors beyond 150× are achieved.

### Compression and decompression speed

To be attractive for large datasets, a compression method should enable compression as well as decompression at reasonable speeds. ECMWF produces data at ~2 GB s^−1^, including CAMS, which creates ~15 MB s^−1^. Data on ECMWF’s archive are compressed once, but downloaded, on average, at 120 MB s^−1^ by different users, such that both high compression and decompression speeds are important. The (de)compression speeds obtained here are all at least 100 MB s^−1^ single-threaded (Supplementary Fig. 9), but faster speeds are available in exchange for lower compression factors (Methods). The real information is only analyzed once and is ultimately independent of the compressor choice.

### A Turing test for data compression

In numerical weather predictions, progress in the development of global weather forecasts is often assessed using a set of error metrics, summarized in so-called score cards. These scores cover important variables in various large-scale regions, such as surface temperature over Europe or horizontal wind speed at different vertical levels in the Southern Hemisphere. With a similar motivation as in ref. ^54^, we suggest assessing the efficiency of climate data compression using similar scores, which have to be passed similarly to a Turing test^34,55^. The compressed forecast data should be indistinguishable from the uncompressed data, or at least the current compression method, while allowing higher compression factors.

Many score tests currently in use represent area averages (such as Fig. 6d), which would also be passed with coarse-grained data. Reducing the horizontal resolution from 10 km to 20 km, for example, yields a compression factor of 4×. It is therefore important to include resolution-sensitive score tests such as the maximum error in a region. Although a compression method either passes or fails such a data compression Turing test, there is additional value in conducting such a test. Evaluating the failures will highlight problems and evaluating the passes may identify further compression potential.

## Discussion

The definition of the bitwise real information content presented here is based on the mutual information in adjacent grid points. We therefore assume a spatial and temporal coherence of data that will come with some autocorrelation. For vanishing autocorrelation in the data the real information content will drop to zero, as the mutual information between bits in adjacent but independent grid points approaches zero. In this case, the entire dataset is identified as false information and consequently rounded to zero. In practice, this only occurs with data having autocorrelation coefficients of less than 0.2 (Supplementary Fig. 4). If there is valuable scientific information in seemingly random data, then the assumption that the mutual information in adjacent grid points is real information does not hold.

Issues with the bitwise real information content can arise in data that was previously subject to lossy compression. Linear or logarithmic quantization, for example, rounds data in linear or logarithmic space, respectively, which is not equivalent to binary rounding in the floating-point format. Consequently, such a quantization will generally introduce non-zero bits in the mantissa of floats when decompressed. These bits can have some statistical dependence, appearing as artificial information induced by the quantization. Such artificial information can be observed as small background information (that is, significantly different from 0) or re-emerging information in the last mantissa bits. In this case, the information distribution across bit positions deviates clearly from the typical (Fig. 2) for which the information drops monotonically with increasing bit position in the mantissa and is insignificantly different from 0 thereafter.

A solution to this quantization-induced artificial information is to apply bitwise real information analysis in the compressed encoding. The bitwise real information content, as defined here, is independent of the binary number format, so it can also be applied to integers representing compressed data from quantization. In our case, rounding in the floating-point representation guarantees that the rounded mantissa bits have zero entropy and therefore zero information. No artificial information is therefore introduced and applying the rounding for floats repeatedly has no effect beyond the first application (idempotence).

No additional uncertainty measure has to be assumed for the distinction of real and false information presented here. The uncertainty of a variable represented in a data array is directly obtained from the distribution of the data itself. Most lossy compression techniques leave the choice of precision to the user, which may lead to subjective choices or the same precision for a group of variables. Instead, our suggestion that 99% of information should be preserved may be altered by the user, which will implicitly determine the required precision for each variable individually.

Lossy compression inevitably introduces errors compared to the uncompressed data. Weather and climate forecast data, however, already contain uncertainties that are, in most cases, larger than the compression error. For example, limiting the precision of the surface temperature to 0.1 °C (as shown in Fig. 3b) is well below the average forecast error (Fig. 6d) and also more precise than the typical precision of 1 °C presented to end users of a weather forecast. Reducing the precision to the real information content not only increases compressibility but also helps to directly communicate the uncertainty within the dataset. The uncertainty of data is important—often neglected—information in itself.

Satisfying the requirements for size, precision and speed simultaneously is an inevitable challenge of data compression. As the precision can be reduced without losing information, we revisit this trade-off and propose an information-preserving compression. At the same time as current archives probably use large capacities to store random bits, analysis of the bitwise real information content is essential for achieving efficient climate data compression.

## Methods

### Data

The CAMS data were analyzed for one time step on 1 December 2019 12:00 ut and bilinearly regridded onto a regular 0.4° × 0.4° longitude–latitude grid using Climate Data Operators (CDO) v1.9. All 137 vertical model levels were included. Global fields of temperature from ECMWF’s ensemble prediction system with 91 vertical levels were used from the first 25 members of a 50-member 15-day ensemble forecast starting on 24 September 2020 at 0:00 ut. Bilinear regridding onto a regular 0.2° × 0.2° longitude–latitude grid was applied (in a similar manner as for the CAMS data). All compression methods here include the conversion from Float64 to Float32.

Only longitude–latitude grids are considered in this Article. However, the methodology can be applied to other grids too. For example, ECMWF’s octahedral grid collapses the two horizontal dimensions into a single horizontal dimension that circles on latitude bands around the globe starting at the South Pole until reaching the North Pole^56^. The fewer grid points of the octahedral grid reduce the size, but the correlation in the latitudinal direction cannot be exploited.

### Bit pattern entropy

An *n*-bit number format has 2^*n*^ bit patterns available to encode a real number. For most data arrays, not all bit patterns are used at uniform probability. The bit pattern entropy is the Shannon information entropy *H*, in units of bits, calculated from the probability of each bit pattern *p*_*i*_:

1 $${H} = \mathop {\sum }\limits_{i = 1}^{2^n} {p_i}{\log _2}{p_i}$$

The bit pattern entropy is *H* ≤ *n* and maximized to *n* bits for a uniform distribution. The free entropy *H*_f_ is the difference *n* − *H*.

### Grid definitions

The compression methods described here were applied to gridded binary data. Data on structured grids can be represented as a tensor, such that for two dimensions the data can be arranged in a matrix *A* with elements *a*_*ij*_ and indices *i*, *j*. Adjacent elements in *A*, for example *a*_*ij*_ and *a*_*i*+1*,j*_, are also adjacent grid points. Every element *a*_*ij*_ is a floating-point number or, in general, a number represented in any binary format. The *n* bits in *a*_*ij*_ are described as bit positions, including sign, exponent and mantissa bits. In the following we will consider sequences of bits that arise from incrementing the indices *i* or *j* while holding the bit position fixed, for example, the sequence of bits consisting of the first mantissa bit in *a*_*ij*_, then the first mantissa bit in *a*_*i*+1,*j*_, and so on. We can refer to these bits as bits from adjacent grid points. Every bit position in elements of *A* is itself a matrix, for example, the matrix of sign bits across all grid points.

### Real information content

The Shannon information entropy^20^ *H* in units of bits takes for a bitstream *b* = *b*_1_*b*_2_ ... *b*_*k*_ ... *b*_*l*_, that is, a sequence of bits of length *l*, the form

2 $${H} = {- {p_0}{\log _2}{p_0}} - {{p_1}{\log _2}{p_1}}$$

with *p*_0_, *p*_1_ being the probability of a bit *b*_*k*_ in *b* being 0 or 1. The entropy is maximized to 1 bit for equal probabilities ${p_0} = {p_1} = {\frac{1}{2}}$ in *b*. We derive the mutual information^41–43^ of two bitstreams *r* = *r*_1_*r*_2_ ... *r*_*k*_ ... *r*_*l*_ and *s* = *s*_1_*s*_2_ ... *s*_*k*_ ... *s*_*l*_. The mutual information is defined via the joint probability mass function *p*_*rs*_, which here takes the form of a 2 × 2 matrix

3 $${{p}_{rs}} = \left( {\begin{array}{*{20}{c}} {p_{00}} & {p_{01}} \\ {p_{10}} & {p_{11}} \end{array}} \right)$$

with *p*_*ij*_ being the probability that the bits are in the state *r*_*k*_ = *i* and *s*_*k*_ = *j* simultaneously and *p*_00_ + *p*_01_ + *p*_10_ + *p*_11_ = 1. The marginal probabilities follow as column- or row-wise additions in *p*_*rs*_, for example, the probability that *r*_*k*_ = 0 is *p*_*r*__=0_ = *p*_00_ + *p*_01_. The mutual information *M*(*r*,*s*) of the two bitstreams *r*, *s* is then

4 $${M(r,s)} = {\mathop {\sum }\limits_{r = 0}^1} {\mathop {\sum }\limits_{s = 0}^1} {p_{rs}}{\log _2}{\left( {\frac{{{p_{rs}}}}{{p_{r = r}p_{s = s}}}} \right)}$$

We now consider the two bitstreams *r*, *s* being the preceding and succeeding bits (for example, in space or time) in a single bitstream *b*, that is, *r* = *b*_1_*b*_2_ ... *b*_*l*__−1_ and *s* = *b*_2_*b*_3_ ... *b*_*l*_. As explained in the section ‘Grid definitions’, this can, for example, be the bitstream of all first mantissa bits in the gridded data. Considering *r*, *s* as the preceding and succeeding bits is equivalent to the bitwise mutual information in adjacent grid points. The (unconditional) entropy is then effectively *H* = *H*(*r*) = *H*(*s*) as in equation (2) and for *l* being very large. The conditional entropies *H*_0_, *H*_1_ are conditioned on the state of the preceding bit *b*_*j*__−1_ being 0 or 1, respectively:

5 $${\begin{array}{l}{H_0} = - {p_{00}}{\log _2}{p_{00}} - {p_{01}}{\log _2}{p_{01}}\\ {H_1} = - {p_{10}}{\log _2}{p_{10}} - {p_{11}}{\log _2}{p_{11}}\end{array}}$$

The conditional entropy is maximized to 1 bit for bitstreams where the probability of a bit being 0 or 1 does not depend on the state of the preceding bit, which is here defined as false information. With the conditional and unconditional entropies and *p*_0_, *p*_1_ as in equation (2) the mutual information *M* of succeeding bits can be written as

6 $${I} = {{H} - {p_0}{H_0} - {p_1}{H_1}}$$

which is the real information content *I*. This definition is similar to that in ref. ^22^, but avoids an additional assumption of an uncertainty measure. Their formulation similarly uses the state of bits as predictors but assesses the conditional probability mass function (p.m.f.) of a dynamical system as predictands. The binwidth of the p.m.f. is chosen to represent the uncertainty in the system, on which the bitwise real information strongly depends. The formulation here avoids such an additional assumption of uncertainty, as bits are used as both predictors and predictands in the conditional entropy. Consequently, the uncertainty is obtained from the data itself solely based on the mutual information between bits in adjacent grid points.

Equation (6) defines the real information as the entropy minus the false information. For bitstreams with either *p*_0_ = 1 or *p*_1_ = 1 (that is, all bits are either 0 or 1), the entropies are zero, *H* = *H*_0_ = *H*_1_ = 0, and we may refer to the bits in the bitstream as being unused. In the case where *H* > *p*_0_*H*_0_ + *p*_1_*H*_1_, the preceding bit is a predictor for the succeeding bit, which means that the bitstream contains real information (*I* > 0).

### The multidimensional real information content

The real information content *I*_*m*_ for an *m*-dimensional array *A* is the sum of the real information along the *m* dimensions. Let *b*_*j*_ be a bitstream obtained by unraveling a given bit position in *A* along its *j*th dimension. Although the unconditional entropy *H* is unchanged along the *m* dimensions, the conditional entropies *H*_0_, *H*_1_ change as the preceding and succeeding bit is found in another dimension; for example, *b*_2_ is obtained by reordering *b*_1_. *H*_0_(*b*_*j*_) and *H*_1_(*b*_*j*_) are the respective conditional entropies calculated from bitstream *b*_*j*_. Normalization by 1/*m* is applied to *I*_*m*_ such that the maximum information is 1 bit in $I_m^ \ast$:

7 $${I{_m^\ast}} = - {{\frac{{p_0}}{m}}{\mathop {\sum }\limits_{j = 1}^{m}} {H_0}{(b_j)}} - {{\frac{{p_1}}{m}}{\mathop {\sum }\limits_{j = 1}^{m}} {H_1}{(b_j)}}$$

Owing to the presence of periodic boundary conditions for longitude, a succeeding bit might be found across the bounds of *A*. This simplifies the calculation as the bitstreams are obtained from permuting the dimensions of *A* and subsequent unraveling into a vector.

### Preserved information

We define the preserved information in a bitstream *s* when approximating *r* (for example, after a lossy compression) via the symmetric normalized mutual information

8 $${R(r,s)} = {\frac{{2M(r,s)}}{{{H(r)} + {H(s)}}}}$$

where *R* is the redundancy of information of *r* in *s*. The preserved information *P* in units of bits is then the redundancy-weighted real information *I* in *r*:

9 $${P(r,s)} = {R(r,s)I(r)}$$

The information loss *L* is 1 − *P* and represents the unpreserved information of *r* in *s*. In most cases we are interested in the preserved information of an array *X* = (*x*_1_, *x*_2_, ..., *x*_*q*_, ..., *x*_*n*_) of bitstreams *x*_*q*_ when approximated by a previously compressed array *Y* = (*y*_1_, *y*_2_, ..., *y*_q_, ..., *y*_*n*_). For an array *A* of floats with *n* = 32 bits, for example, *x*_1_ is the bitstream of all sign bits unraveled along a given dimension (for example, longitudes) and *x*_32_ is the bitstream of the last mantissa bits. The redundancy *R*(*X*, *Y*) and the real information *I*(*X*) is then calculated for each bit position *q* individually. The fraction of preserved information *P* is the information-weighted mean of the redundancy:

10 $${P(X,Y)} = {\frac{{{\mathop {\sum }\nolimits_{q = 1}^{n}} {R({x_q},{y_q})I({x_q})}}}{{{\mathop {\sum }\nolimits_{q = 1}^{n}} {I({x_q})}}}}$$

The quantity ${{\mathop {\sum}\nolimits_{q = 1}^{n}} {{I({x_q})}}}$ is the total information in *X* and therefore also in *A*. The redundancy is *R* = 1 for bits that are unchanged during rounding and *R* = 0 for bits that are rounded to zero. The preserved information with bitshave or halfshave^29,30^ (that is, replacing mantissa bits without real information with either 00…00 or 10…00, respectively) is therefore equivalent to truncating the bitwise real information for the (half)shaved bits. For round-to-nearest, however, the carry bit depends on the state of bits across several bit positions. To account for the interdependency of bit positions, the mutual information has to be extended to include more bit positions in the joint probability *p*_*rs*_, which will then be a *m* × 2 matrix. For computational simplicity, we truncate the real information as the rounding errors of round-to-nearest and halfshave are equivalent.

### Significance of real information

In the analysis of real information it is important to distinguish between bits with very little but significant information and those with information that is insignificantly different from zero. Although the former have to be retained, the latter should be discarded to increase compressibility. A significance test for real information is therefore presented.

For an entirely independent and equal occurrence of bits in a bitstream of length *l*, the probabilities *p*_0_, *p*_1_ of a bit being 0 or 1 approach ${{p_0} = {p_1} = {\frac{1}{2}}}$, but they are in practice not equal for *l* < ∞. Consequently, the entropy is smaller than 1, but only insignificantly. The probability *p*_1_ of successes in the binomial distribution (with parameter *p* = ½) with *l* trials (using the normal approximation for large *l*) is

11 $${{p_1} = {\frac{1}{2}} + {\frac{z}{{2\sqrt l }}}}$$

where *z* is the ${{1} - {{\frac{1}{2}}{(1 - c)}}}$ quantile at confidence level *c* of the standard normal distribution. For *c* = 0.99, corresponding to a 99% confidence level, which is used as default here, *z* = 2.58, and for *l* = 5.5 × 10^7^ (the size of a 3D array from CAMS), a probability ${{{\frac{1}{2}} \le {p} \le {p_1}} = {0.5002}}$ is considered insignificantly different from equal occurrence *p*_0_ = *p*_1_. The associated free entropy *H*_f_ in units of bits follows as

12 $${{H_{\rm{f}}} = {{1} - {{p_1}{\log _2}{p_1}} - {{({1} - {p_1})}{\log _2}{({1} - {p_1})}}}}$$

We consider real information below *H*_f_ as insignificantly different from 0 and set the real information *I* = 0.

### Dependency of the bitwise real information on correlation

The real information as defined here depends on the mutual information of bits in adjacent grid points. Higher autocorrelation in data (meaning a higher correlation between adjacent grid points) increases the mutual information in the mantissa bits. With higher correlation, the adjacent grid values are closer, increasing the statistical dependence of mantissa bits that would otherwise be independent at lower correlation. Consequently, the real bitwise information content is increased and more mantissa bits have to be retained to preserve 99% of real information (Supplementary Fig. 4a,b).

The increasing number of retained mantissa bits with higher autocorrelation in data will decrease the compression factors, as it is easier to compress bits that are rounded to zero. However, a higher correlation also increases the redundancy in bits of adjacent grid points, which favors a more efficient lossless compression. These two effects counteract, and compression factors only increase piecewise over a small range of correlations while the retained mantissa bits are constant (Supplementary Fig. 4c,d). Once an additional mantissa bit has to be retained to preserve 99% of real information, the compression factors jump back down again, resulting in a sawtooth wave. Over a wide range of typical correlation coefficients (0.5–0.9999) the compression factors are otherwise constant and no higher compressibility is found with increased correlation.

The compression factors can, however, depend on the range of values represented in binary. A shift in the mean to have positive or negative values only means that the sign bit is unused, which increases compression factors (compare Supplementary Fig. 4a,b), despite identical correlation coefficients. Although the correlation is invariant under multiplicative scaling and addition, the bitwise information changes under addition. When the range of values in data fits into a power of two, its real information is shifted across bit positions into the mantissa bits, such that the exponent bits are unused. This can be observed for atmospheric temperatures stored in kelvin (within 200–330 K) where only the last exponent bit and mantissa bits contain information (Supplementary Fig. 10). Using celsius instead shifts information from the mantissa bits into the exponent and sign bits.

### Preservation of gradients

The preservation of gradients and other higher-order derivatives in data is a challenging aspect of compression. Removing false information in data via rounding can result in identical values in adjacent grid points. Even if these values were not identical before rounding, they may not be significantly different from each other in the sense of real and false information. In this case, a previously weak but non-zero gradient will be rounded to zero, which also reduces the variance locally. In other cases, the rounding error is small compared to the standard deviation of the data, such that rounding has a negligible impact on the variance, as values are independently equally likely to be rounded up or down.

The preservation of gradients is illustrated in the example of analyzing oceanic fronts obtained from satellite measurements of sea surface temperatures (Supplementary Fig. 11). Identified by large horizontal gradients in temperature, the location and strength of oceanic fronts is well preserved using compressed data. However, areas of very weak gradients can largely vanish with round + lossless. In this case the temperatures in adjacent grid points are insignificantly different from each other and therefore the gradient is zero after the removal of false information. Weak gradients are better preserved with Zfp compression at similar compression factors, but its block structure becomes visible.

### Rounding

With round-to-nearest, a full-precision number is replaced by the nearest representable float with fewer mantissa bits by rounding the trailing bits to zero. Representing *π* as the 32-bit float *f*, for example, can then be rounded to six mantissa bits as

13 $${\begin{array}{rcl}f &=& {0}\,{10000000}\,{10010010000111111011011} = 3.1415927\\\mathrm{round}\left(f\right) &=& {0}\,{10000000}\,{100101}{00000000000000000} = {3.15625}\end{array}}$$

The 32 bits are split into sign, 8 exponent bits and 23 mantissa bits. The sixth mantissa bit flips due to the carry bit; that is, *f* is rounded up, *f* < round(*f*). Alternative rounding modes have been proposed for data compression^29,30^, but many suffer from some bias or introduce larger rounding errors.

### Error norms

The normalized absolute error ${{E_{\rm{abs}}^{\ast}}}$ of an element ${\bar a}$ from a compressed array ${\bar A}$ relative to the respective element *a* from full-precision array *A* is

14 $${{{E_{\rm{abs}}^ \ast}} = {\frac{{\left| {{\bar a} - {a}} \right|}}{{{{{{\mathrm{mean}}}}(\left| A \right|)}}}}}$$

where ${\left| A \right|}$ denotes the element-wise absolute value of *A*. The normalization with ${{{{\mathrm{mean}}}}{(\left| A \right|)}}$ is therefore the same for all element pairs across *A* and ${\bar A}$, which distinguishes it from a relative error. It is used to make the absolute errors between variables with different value ranges comparable. The expected error in the mean is zero with the bias-free rounding mode round-to-nearest and therefore the mean error is not explicitly analyzed here. Zfp compression can, however, introduce small errors in the mean^57,58^. The decimal error *E*_dec_ is^59^

15 $${{{E}_{\rm{dec}}} = {\left| {{{{{\mathrm{log}}}}_{10}}{\left( {\frac{{\bar a}}{a}} \right)}} \right|}}$$

Special cases are *E*_dec_ = ∞ when *a* or ${\bar a}$ is 0 or the signs do not match, ${{{\rm{sign}}{(a)}} \ne {{\rm{sign}}{(\bar a)}}}$, unless ${{\bar a} = {a} = {0}}$ in which case *E*_dec_ = 0. The decimal error is used to better highlight when lossy data compression changes the sign (with sign(0) = 0) of a value. Bounding the absolute or relative error does not enforce that. The maximum normalized absolute and decimal errors are then the maximum of all ${{{E}_{\rm{abs}}^ {\ast}}}$ and *E*_dec_, respectively, computed for all element pairs across *A* and ${\bar A}$. The rounding in the round + lossless method does not affect the sign or the exponent bits, such that the probability of sign changes is zero.

### Structural similarity

A metric to assess the quality of lossy compression in image processing is the structural similarity index measure (SSIM)^60^. For images it is based on comparisons of luminance, contrast and structure. For floating-point arrays the luminance contributions to SSIM can be interpreted as the preservation of the mean, and the contrast compares the variances and the structure compares the correlation. The SSIM of two arrays *A*, *B* of the same size is defined as

16 $${{{\rm{SSIM}}{(A,B)}} = {\frac{{{{\left( {{2{\mu _A}{\mu _B}} + {c_1}} \right)}{\left( {{2{\sigma _{AB}}} + {c_2}} \right)}}}}{{{{\left( {{\mu _A^2} + {\mu _B^2} + {c_1}} \right)}{\left( {{\sigma _A^2} + {\sigma _B^2} + {c_2}} \right)}}}}}}$$

where *μ*_*A*_, *μ*_*B*_ are the respective means, ${{\sigma _A^2},{\sigma _B^2}}$ the respective variances and *σ*_*AB*_ the covariance. *c*_1_ = (*k*_1_*L*)^2^ and *c*_2_ = (*k*_2_*L*)^2^ are introduced to increase stability with a small denominator and *k*_1_ = 0.01 and *k*_2_ = 0.03. The dynamic range is *L* = max(max(*A*), max(*B*)) − min(min(*A*), min(*B*)). The SSIM is a value in [0, 1] where the best possible similarity SSIM = 1 is only achieved for identical arrays *A* = *B*.

For rounded floating-point arrays the decimal error is proportional to the square root of the dissimilarity, 1 − SSIM (Supplementary Fig. 5c). The SSIM in this case is approximately equal to the correlation, as round-to-nearest is bias-free (that is, *μ*_*A*_ ≈ *μ*_*B*_) and the rounding error is typically much smaller than the standard deviation of the data (that is, *σ*_*A*_ ≈ *σ*_*B*_). Here, we use the logarithmic SSIM, SSIM_log_(*A*, *B*) = SSIM(log*A*, log*B*), which is the SSIM applied to log-preprocessed data (the logarithm is applied element-wise). The usage of SSIM_log_ is motivated by the rather logarithmic data distribution for most variables (Supplementary Fig. 1), but similar results are obtained for SSIM. The proportionality to the decimal error is unchanged when using SSIM_log_.

Baker et al. proposed the SSIM as a quality metric for lossy compression of climate data^54^. Although for image processing SSIM > 0.98 is considered good quality, Baker et al. suggest a higher threshold of SSIM = 0.99995 for climate data compression. The preserved information as defined here can be used as a compression quality metric similar to the SSIM. When preserving 99% of real information, the SSIM_log_ is also above the Baker threshold (Supplementary Fig. 5b), reassuring us that our threshold of 99% preserved real information is reasonable. In general, the preserved information is a monotonic function of the structural similarity SSIM (or SSIM_log_) for rounded floating-point arrays, further supporting the usage of preserved information as a metric for data compression.

### Linear and logarithmic quantization

The *n*-bit linear quantization compression for each element *a* in an array *A* is

17 $${{\bar a} = {{{{{\mathrm{round}}}}}{\left( {{{2}^{n - 1}}{\frac{{{a} - {\min (A)}}}{{{{{{\mathrm{max}}}}(A)} - {{{{\mathrm{min}}}}(A)}}}}} \right)}}}$$

with round a function that rounds to the nearest integer in 0, ..., 2^*n* − 1^. Consequently, every compressed element $\bar a$ can be stored with *n* bits. The *n*-bit logarithmic quantization compression for every element $a \ge 0$ in *A* is

18 $${{\bar a} = {\left\{ {\begin{array}{*{20}{l}} {0} \hfill & {{{{\mathrm{if}}}}\, {a = 0}} \hfill \\ {{{{\mathrm{round}}}}{({c} + {{{\varDelta}^{ - 1}}{\log a}})} + {1}} \hfill & {{{{\mathrm{else}}}}} \hfill \end{array}} \right.}}$$

to reserve the zero bit pattern 0…0 to encode 0. The logarithmic spacing is

19 $${{\varDelta} = {\frac{{{\log \left( {\max \left( A \right)} \right)} - {\log \left( {{{{{\mathrm{min}}}}^ +} {\left( A \right)}} \right)}}}{{{2^n} - {2}}}}}$$

The constant ${{c} = {{\frac{1}{2}} - {{{\varDelta}^{ - 1}}{\log {\left( {\frac{{{{{{\mathrm{min}}}}^ +}{ \left( A \right)}}}{2}{({\rm{e}}^{{{{\varDelta}}}} + {1})}} \right)}}}}}$ is chosen to implement round-to-nearest in linear space instead of in logarithmic space, for which ${{c} = - {{\varDelta}^{ - 1}}{\log \left( {{{{{\mathrm{min}}}}^ +} {\left( A \right)}} \right)}}$. The function min^+^(*A*) is the minimum of all positive elements in *A*.

### Lossless compression

We use Zstandard as a default lossless algorithm for the round + lossless method. Zstandard is a modern compression algorithm that combines many techniques to form a single compressor with tunable 22 compression levels that allow large trade-offs between compression speed and factors^47^^,^^50^. Here we use compression level 10, as it presents a reasonable compromise between speed and size. Zstandard outperforms other tested algorithms (deflate, LZ4, LZ4HC and Blosc) in our applications and is also found to be among the best in the lzbench compression benchmark^47^ and other studies have focused on comparisons^45^. Lossless compressors are often combined with reversible transformations that preprocess the data. The so-called bitshuffle^45^ transposes an array on the bit-level, such that bit positions (for example, the sign bit) of floating-point numbers are stored next to each other in memory. Another example is the bitwise XOR operation^61^ with the preceding floating-point value, which sets subsequent bits that are identical to 0. Neither bitshuffle nor XOR notably increased the compression factors in our applications.

### Matching preserved bits to the precision of Zfp

The Zfp compression algorithm divides a *d*-dimensional array into blocks of size 4^*d*^ to exploit correlation in every dimension of the data. Within each block, a transformation of the data is applied with specified absolute error tolerance or precision, which bounds a local relative error. We use Zfp in its precision mode, which offers discrete levels to manually adjust the retained precision. Owing to the rather logarithmic distribution of CAMS data (Supplementary Fig. 1), a log-preprocessing of the data is applied to prevent sign changes (including a flushing to zero) within the compression^57,58^. The error introduced by Zfp is approximately normally distributed and therefore usually yields higher maximum errors compared to round-to-nearest in float arithmetic, although median errors are comparable. To find an equivalent error level between the two methods, we therefore choose the precision level of Zfp to yield median absolute and decimal errors that are at least as small as those from rounding. The manual choice of the precision level is hence tied to the analysis of the bitwise real information content and automated.

This method is illustrated in Supplementary Fig. 12 in more detail. Errors introduced from round-to-nearest for floats have very rigid error bounds. The majority of errors from Zfp compression are within these bounds when matching median errors. However, given the normal distribution of errors with Zfp, there will be a small share of errors that are beyond the bounds from round-to-nearest. Using the precision mode of Zfp and log-preprocessed data bounds these maximum errors well.

### Compressor performances

Although different compressors and their performance are not within the central focus of this study, we analyze the compression and decompression speeds as a sanity check (Supplementary Fig. 9). To find a data compression method that can be used operationally, a certain minimum data throughput should be achieved. The current 24-bit linear quantization method reaches compression speeds of almost 800 MB s^−1^ single-threaded on an Intel i7 (Kaby Lake) central processing unit in our application, excluding writing to disk. For the logarithmic quantization, this would decrease to ~200 MB s^−1^ due to the additional evaluation of a logarithm for every value. For Zstandard, the user can choose between 22 compression levels, providing a trade-off between the compression speed (highest for level 1) and the compression factor (highest for level 22). The compression speed reduces from ~700 MB s^−1^ at compression level 1 to 2 MB s^−1^ at level 22, such that for high compression factors about 1,000 cores would be required in parallel to compress in real time the 2 GB s^−1^ data production at ECMWF. For Zstandard at compression level 10, speeds of at least 100 MB s^−1^ are achieved, but at the cost of about 50% larger file sizes. We use compression level 10 throughout this study as a compromise. The decompression speed is independent of the level. The additional performance cost of binary rounding is negligible with 2 GB s^−1^. Zfp reaches compression speeds of ~200 MB s^−1^ (single-threaded, including the log-preprocessing) in our application, enough to compress ECMWF’s data production in real time with a small number of processors in parallel.

### Supplementary information

Supplementary Information Supplementary Table 1 and Figs. 1–12.

### Source data

Source Data Fig. 2 Source data as zipped .csv.

Source Data Fig. 3 Source data as zipped .csv.

Source Data Fig. 4 Source data as zipped .csv.

Source Data Fig. 5 Source data as zipped .csv.

Source Data Fig. 6 Source data as zipped .csv.

## Data Availability

The entire CAMS dataset is freely available to download from the Copernicus Atmosphere Data Store at https://atmosphere.copernicus.eu/data. Full precision data that were not subject to lossy compression before, as used here, are available from the Copernicus Atmosphere Monitoring Service^62^ and the European Centre for Medium-Range Weather Forecasts^63^. Source data are provided with this paper.

## Code availability

The software that was developed for this study is available in the published Julia packages BitInformation.jl (v0.2), LinLogQuantization.jl (v0.2) and ZfpCompression.jl (v0.2). All scripts to reproduce the analysis are available in ref. ^64^. Relevant software and a subset of the data are available on Code Ocean^65^
