# Supplementary material for: Compressing atmospheric data into its real information content
Source: Nat Comput Sci. 2021 Nov 25;1(11):713–24. doi: 10.1038/s43588-021-00156-2 (PMC10766530; doi:10.1038/s43588-021-00156-2)
Supplement: Supplementary file 1 — Supplementary Table 1 and Figs. 1–12. [file 43588_2021_156_MOESM1_ESM.pdf]

nature portfolio

https://doi.org/10.1038/s43588-021-00156-2

Supplementary information

Compressing atmospheric data into its real information content

In the format provided by the authors and unedited

Supplementary information

for
M Klöwer, M Razinger, JJ Dominguez, PD Düben and TN Palmer, 2021. *Compressing atmospheric data into its real information content*, **Nature Computational Science**.

Contents

- Table 1, List of atmospheric variables in CAMS
- Figure 1, Statistical distributions of variables in CAMS
- Figure 2, Bitpattern histogram for linear and logarithmic quantization
- Figure 3, Resolution dependence of the information-preserving compression
- Figure 4, Dependency of the bitwise real information and compressibility on correlation
- Figure 5, Preserved information, decimal error and structural similarity
- Figure 6, Compression of radar-based observations of precipitation over Great Britain
- Figure 7, Compression of satellite-based observations of brightness temperature
- Figure 8, Compression of nitrogen dioxide and methane at the surface
- Figure 9, Compressor performances
- Figure 10, Bitwise real information content for temperature in various dimensions
- Figure 11, Preservation of gradients during compression
- Figure 12, Error distribution of binary rounding compared to Zfp compression

Name	Code	Unit	Name	Code	Unit
**Aerosols**			**Carbon oxides**		
Aerosol optical thickness 532nm	aot532	1	Carbon dioxide	co2	kg/kg
Anthropogenic aot532	aaot532	1	Carbon monoxide	co	kg/kg
Natural aot532	naot532	1	**Clouds and water**		
Backscatter from ground at 1064nm	aergnd1064	$m^{-1}sr^{-1}$	Fraction of cloud cover	cc	1
Backscatter from ground at 355nm	aergnd355	$m^{-1}sr^{-1}$	Cloud ice water content	ciwc	kg/kg
Backscatter from ground at 532nm	aergnd532	$m^{-1}sr^{-1}$	Cloud liquid water content	clwc	kg/kg
Backscatter from top of atm at 1064nm	aertoa1064	$m^{-1}sr^{-1}$	Specific rain water content	crwc	kg/kg
Backscatter from top of atm at 532nm	aertoa355	$m^{-1}sr^{-1}$	Specific snow water content	cswc	kg/kg
Backscatter from top of atm at 532nm	aertoa532	$m^{-1}sr^{-1}$	Specific humidity	q	kg/kg
Aerosol extinction coefficient at 1064nm	aerext1064	m^{-1}	**Methane**		
Aerosol extinction coefficient at 355nm	aerext355	m^{-1}	Methane	ch4	kg/kg
Aerosol extinction coefficient at 532nm	aerext532	m^{-1}	Methane (chemistry)	ch4_c	kg/kg
Aerosol type 2 source/gain accumulated	aergn02	kg/m^2	Methane loss rate	kch4	s^{-1}
Aerosol type 7 source/gain accumulated	aergn07	kg/m^2	**Alkanes or alcohols**		
Aerosol type 9 source/gain accumulated	aergn09	kg/m^2	Ethene	c2h4	kg/kg
Aerosol type 10 source/gain accumulated	aergn10	kg/m^2	Ethanol	c2h5oh	kg/kg
Aerosol type 11 source/gain accumulated	aergn11	kg/m^2	Ethane	c2h6	kg/kg
Aerosol large mode mixing ratio	aerlg	kg/kg	Propane	c3h8	kg/kg
Sea salt (0.03-0.5μm)	aermr01	kg/kg	Isoprene	c5h8	kg/kg
Sea salt (0.5-5μm)	aermr02	kg/kg	Acetone	ch3coch3	kg/kg
Sea salt (5-20μm)	aermr03	kg/kg	Methanol	ch3oh	kg/kg
Dust aerosol (0.03-0.55μm)	aermr04	kg/kg	Methyl peroxide	ch3ooh	kg/kg
Dust aerosol (0.55-0.9μm)	aermr05	kg/kg	Hydrogen peroxide	h2o2	kg/kg
Dust aerosol (0.9-20μm)	aermr06	kg/kg	Formaldehyde	hcho	kg/kg
Hydrophilic organic matter	aermr07	kg/kg	Formic acid	hcooh	kg/kg
Hydrophobic organic matter	aermr08	kg/kg	Nitric acid	hno3	kg/kg
Hydrophilic black carbon	aermr09	kg/kg	Hydroperoxy radical	ho2	kg/kg
Hydrophobic black carbon	aermr10	kg/kg	Hydroxyl radical	oh	kg/kg
Sulphate aerosol	aermr11	kg/kg	Aldehyde	ald2	kg/kg
Nitrate fine mode	aermr16	kg/kg	**Nitrogen and sulphur oxides**		
Nitrate coarse mode	aermr17	kg/kg	Nitrogen dioxide	no2	kg/kg
Ammonium aerosol	aermr18	kg/kg	Nitrogen monoxide	no	kg/kg
**Others**			Sulphur dioxide	so2	kg/kg
Olefins	ole	kg/kg	**Ozone**		
Organic nitrates	onit	kg/kg	Ozone mass mixing ratio 2	go3	kg/kg
Peroxyacetyl nitrate	pan	kg/kg	Ozone mass mixing ratio 1	o3	kg/kg
Paraffins	par	kg/kg	Stratospheric ozone	o3s	kg/kg

Supplementary Table 1 | List of atmospheric variables in CAMS with name, variable code and unit.

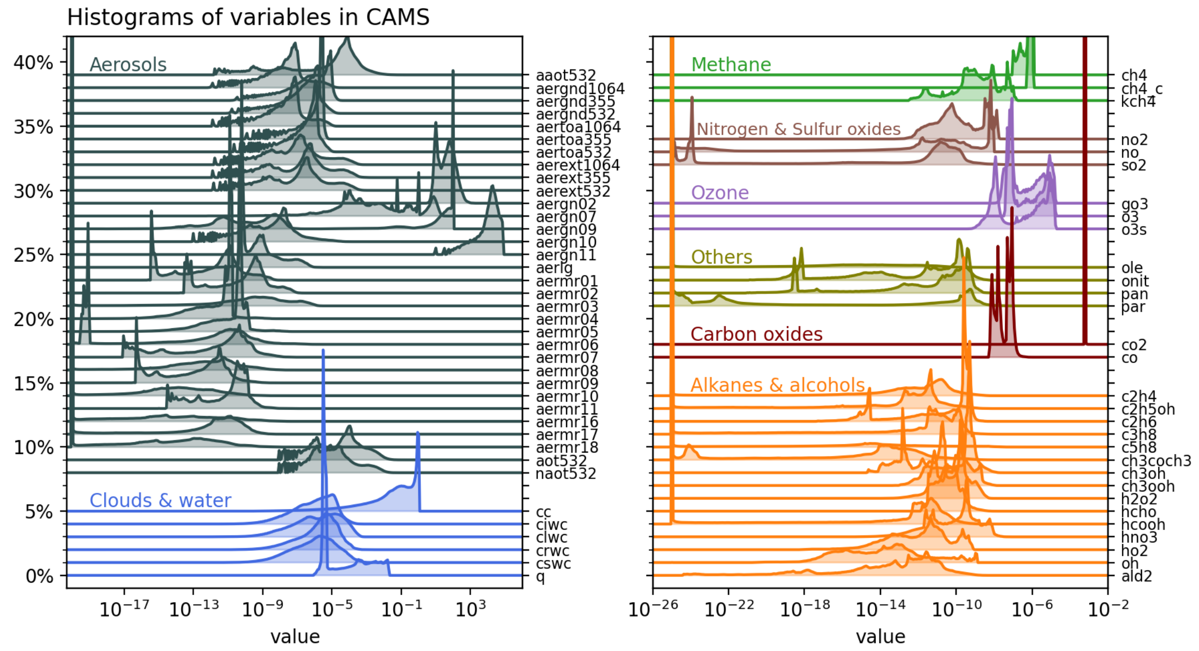

Supplementary Figure 1 | Statistical distributions of all variables in CAMS. Histograms use a logarithmic binning and are staggered vertically for clarity. The variable abbreviations are explained in Table 1.

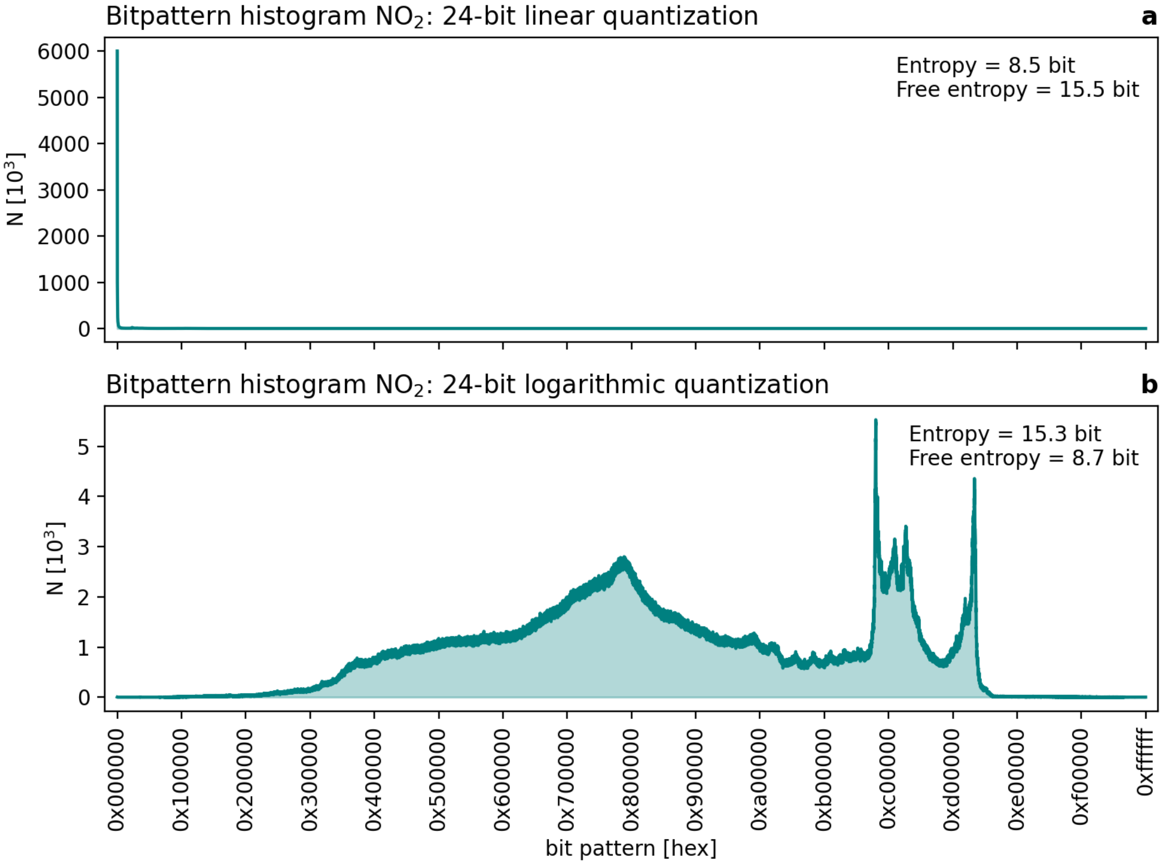

Supplementary Figure 2 | Bitpattern histogram for linear and logarithmic quantization. a linear 24-bit quantization and **b** 24-bit logarithmic quantization of nitrogen dioxide NO_2 mixing ratio [kg/kg]. All grid points and all vertical levels are used, consisting of $5.6 \cdot 10^7$ values with a range of $2 \cdot 10^{-14}$ to $2 \cdot 10^{-7}$ kg/kg. Bitpatterns are denoted in 24-bit hexadecimal. The free entropy is the difference between the available 24 bit and the bitpattern entropy (see Methods) and quantifies the number of effectively unused bits.

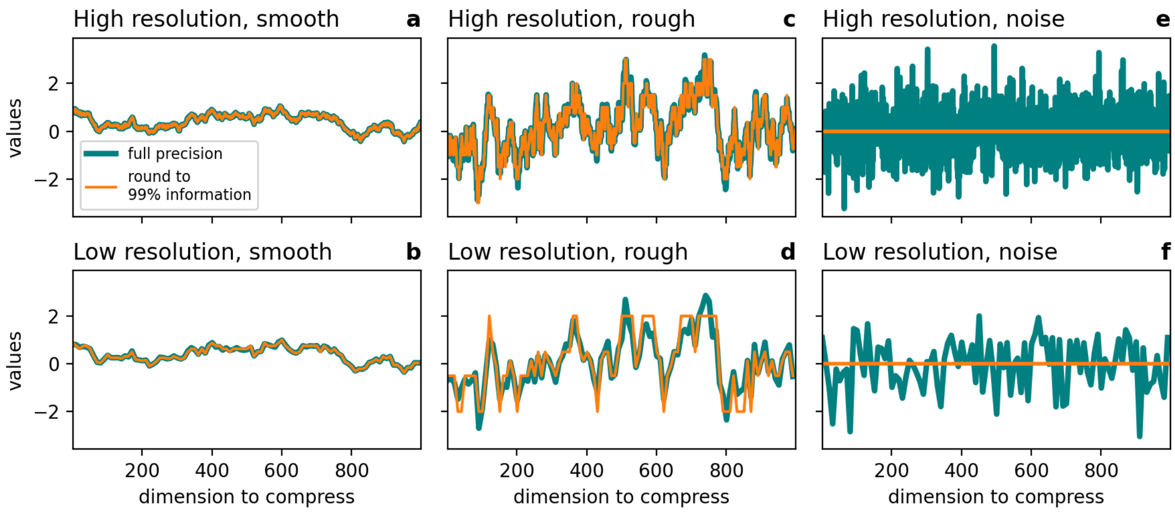

Supplementary Figure 3 | Resolution and smoothness dependence of the information-preserving compression. a,b Highly autocorrelated data (1st order auto-regressive process with correlation r=0.999) will have many mantissa bits preserved, at high and low resolution. **c,d** Many mantissa bits in data with less autocorrelation (r=0.95) will be independent at low resolution and therefore rounded to zero. **e,f** All bits in random data (r=0) drawn from a standard normal distribution are fully independent so that removing the false information rounds this data to zero. Low resolution data (**b,d,f**) is obtained from high resolution (**a,c,e**) by subsampling every 10th data point.

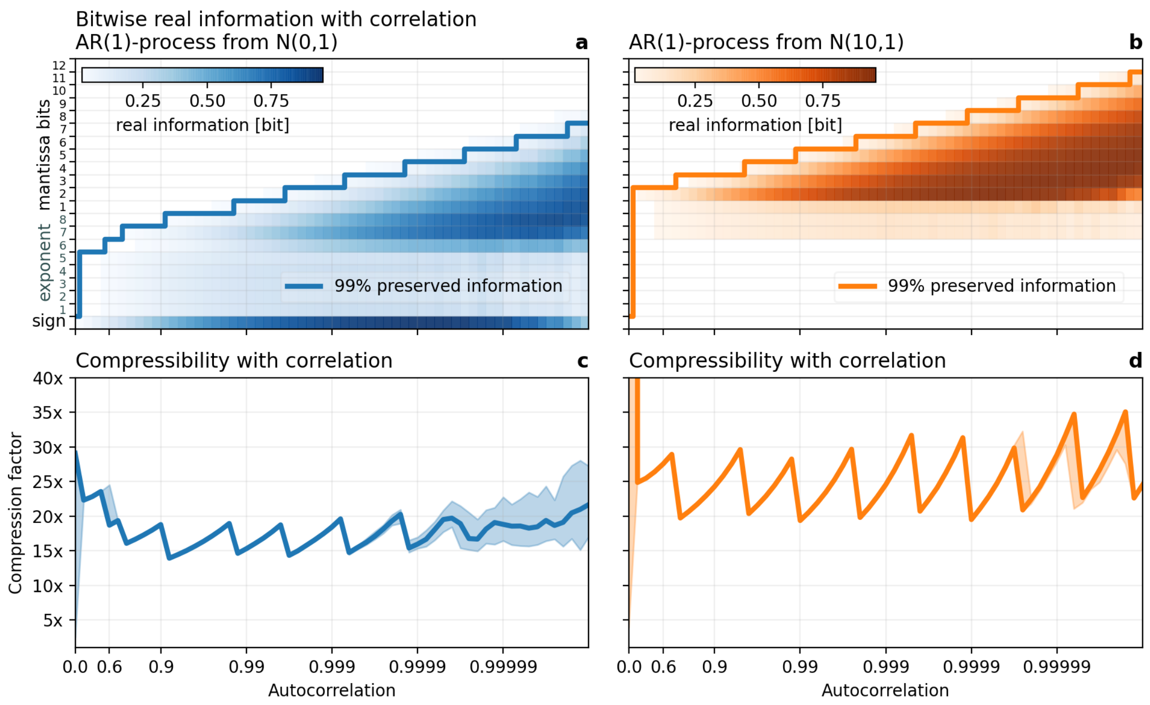

Supplementary Figure 4 | Dependency of the bitwise real information and compressibility on correlation. a The bitwise real information content of a first-order autoregressive process (AR(1) with Gaussian distribution N(0,1), i.e. with zero mean and unit variance) with varying lag-1 autocorrelation. The bits that have to be retained to preserve 99% of information are enclosed with a solid line. **b** as **a** but the AR(1) process follows a Gaussian distribution with a mean of 10. **c,d** Compression factors for **a,b** when preserving 99% of information. Shading denotes the interdecile range.

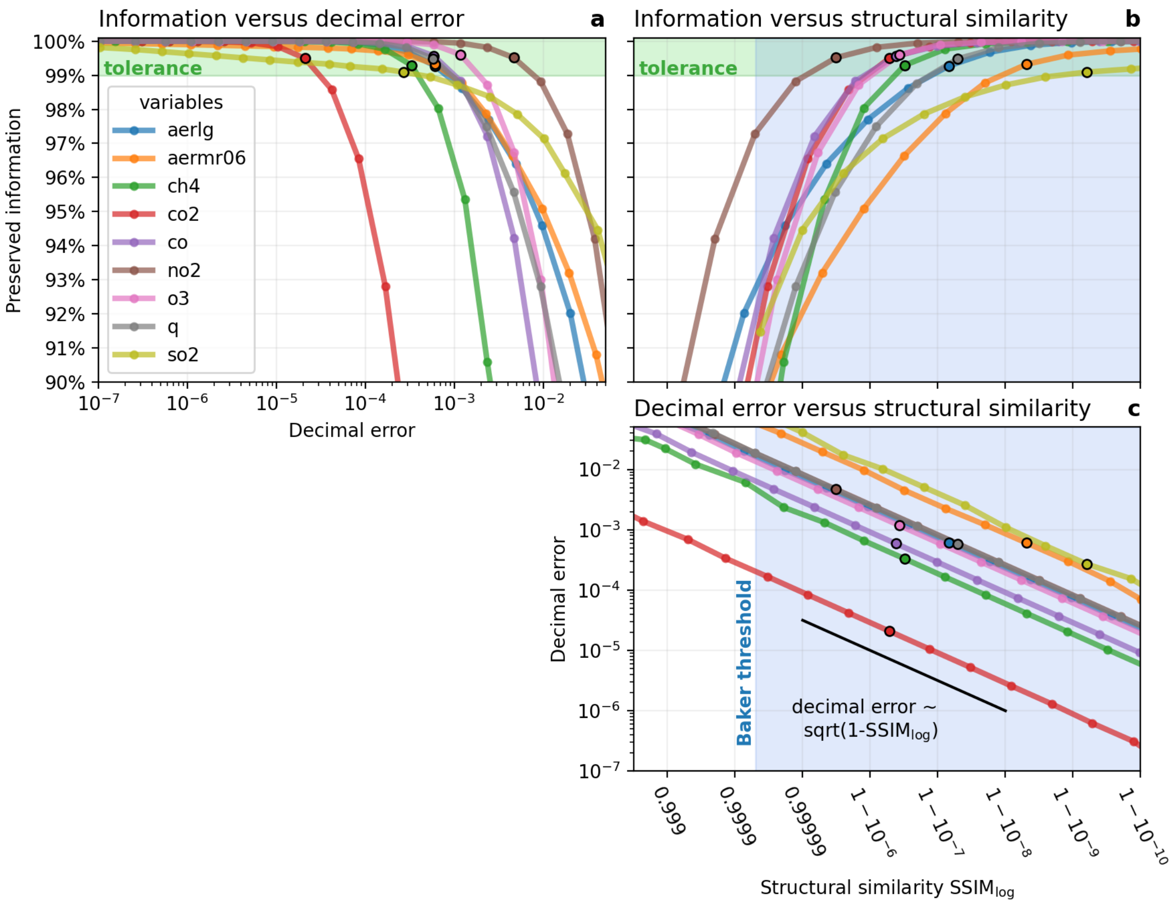

Supplementary Figure 5 | The relationships between preserved information, decimal error and structural similarity for rounding within the information-preserving compression. a The last 1% of information tends to be distributed across many mantissa bits such that a trade-off arises where a large increase in compressibility is achieved for a small tolerance in information loss. The preserved information is a function of the decimal error, which itself increases exponentially for every additional bit (small circles) that is discarded due to rounding. Denoted circles present the number of mantissa bits that have to be retained during compression to preserve at least 99% of information. **b** The preserved information increases as a function of the structural similarity (SSIM)[57]. The proposed threshold for climate data of SSIM=0.99995 by Baker et al. 2019 is shaded[53]. All variables are very close or above the Baker threshold when preserving 99% of information. **c** The decimal error is proportional to the square root of the structural dissimilarity 1-SSIM for binary rounding within the information-preserving compression.

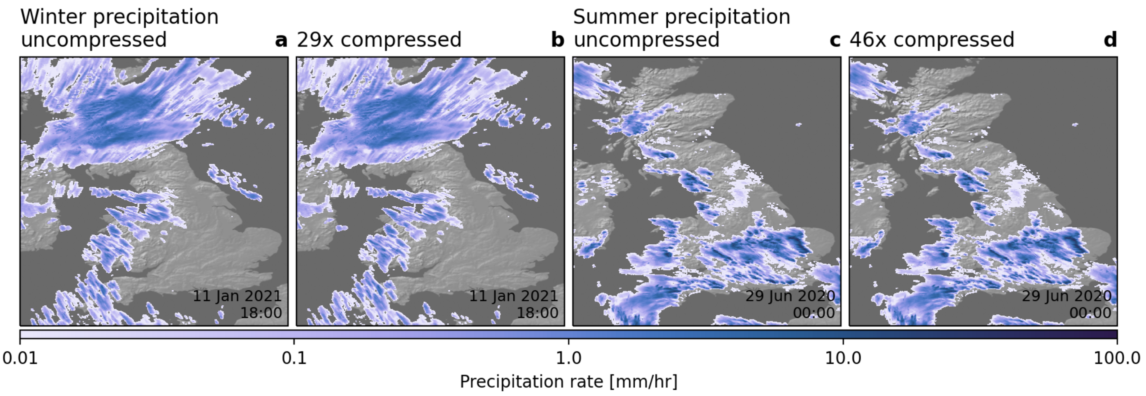

Supplementary Figure 6 | Compression of radar-based observations of precipitation over Great Britain. a Precipitation for the hour preceding 18:00 UTC on 11 Jan 2021 from the UK MetOffice NIMROD data at about 1km horizontal resolution. **b** as **a** but the data was compressed preserving 99% of real information achieving compression factors of 29x relative to 64 bit. **c** and **d** as **a** and **b** but for 00:00 UTC on 29 Jun 2021 and achieving compression factors of 46x. 2 mantissa bits are retained in this data set.

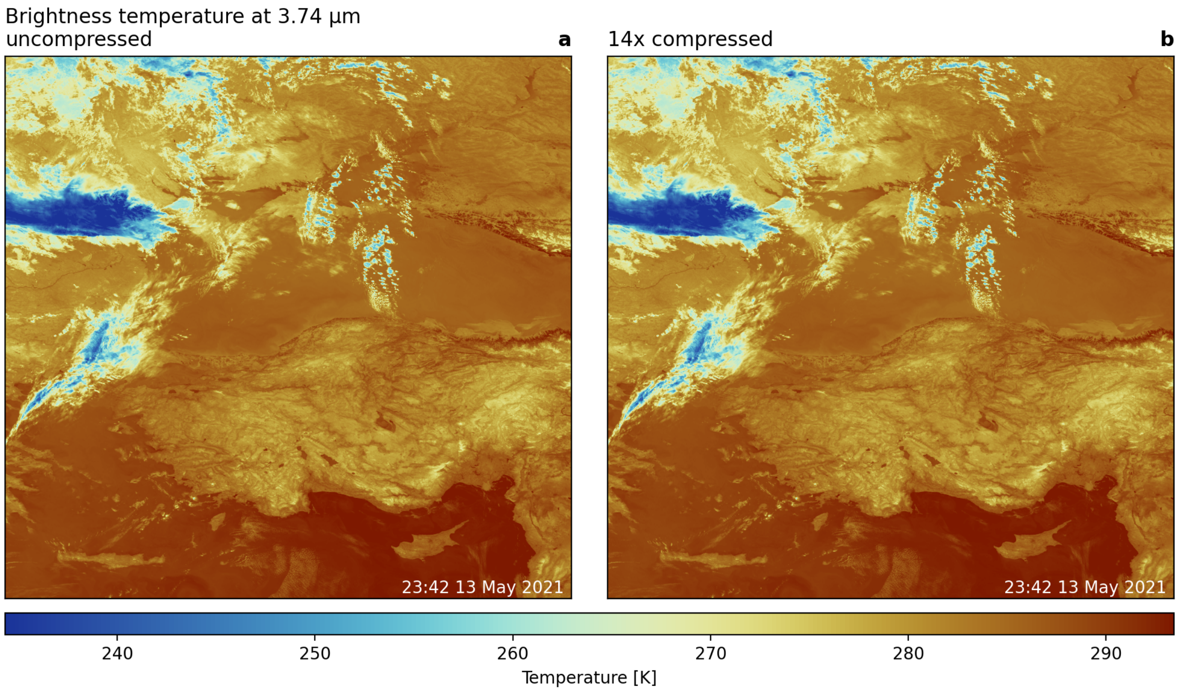

Supplementary Figure 7 | Compression of satellite-based observations of brightness temperature over the Black Sea and Turkey. a Brightness temperature measured by the 3.74µm (I4) channel of the VIIRS sensor on board the Suomi-NPP satellite at about 300m horizontal resolution on the 13 May 2021. **b** as **a** but the data was compressed preserving 99% of real information with the round+lossless method achieving compression factors of 14x relative to 64 bit. 9 mantissa bits are retained in this data set.

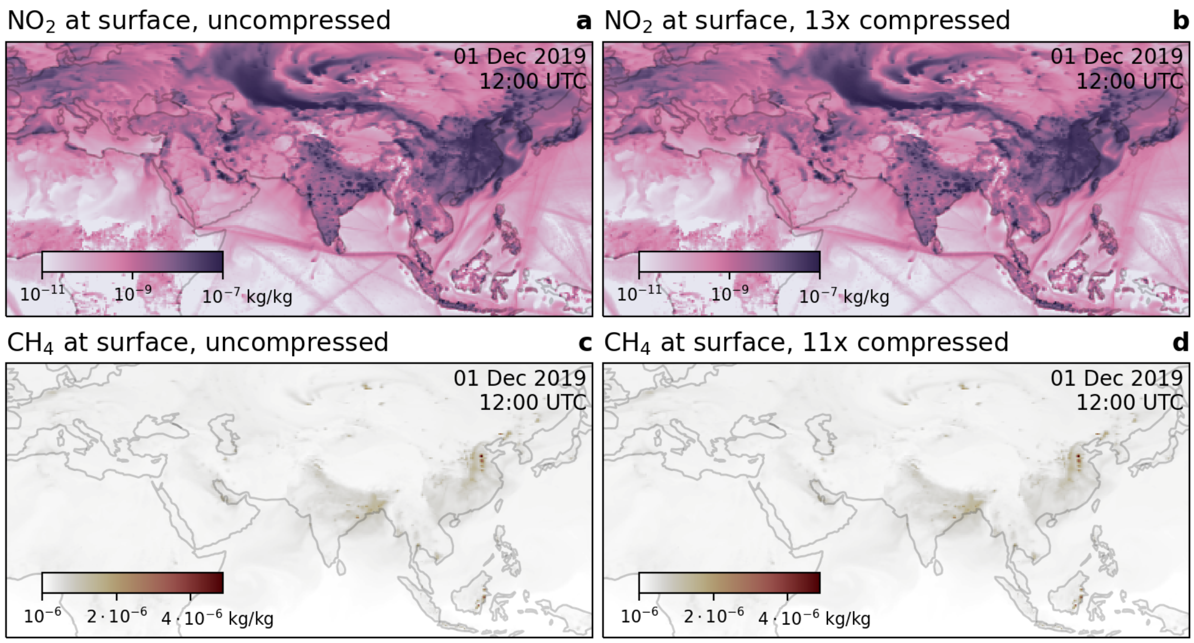

Supplementary Figure 8 | Compression of nitrogen dioxide (NO_2) and methane (CH_4) at the surface. **a** Surface NO_2 concentrations preliminary result from fossil fuel combustion. **b** Surface CH_4 concentrations often include point sources, such as here in East China, East India and East Borneo. **b,d** as **a,c** but compressed preserving 99% of information achieving a compression factor of 13x, 11x. 3 (12) mantissa bits are retained for NO_2 (CH_4) at the surface.

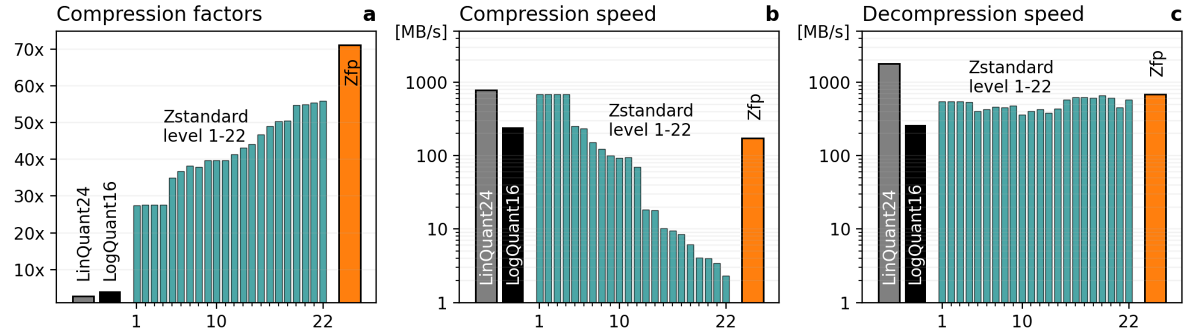

Supplementary Figure 9 | Compressor performances. Compressing water vapour (specific humidity, variable code q) (3 mantissa bits retained, as in Fig. 3) with 24-bit linear quantization (LinQuant24), 16-bit logarithmic quantization (LogQuant16), round+lossless (Zstandard, compression level 1-22) and Zfp (precision-mode, including log-preprocessing): **a** Compression factors, **b** compression speed, **c** decompression speed. Timings are single-threaded on an Intel Core™ i7 (Kaby Lake) and do not include the writing to disk.

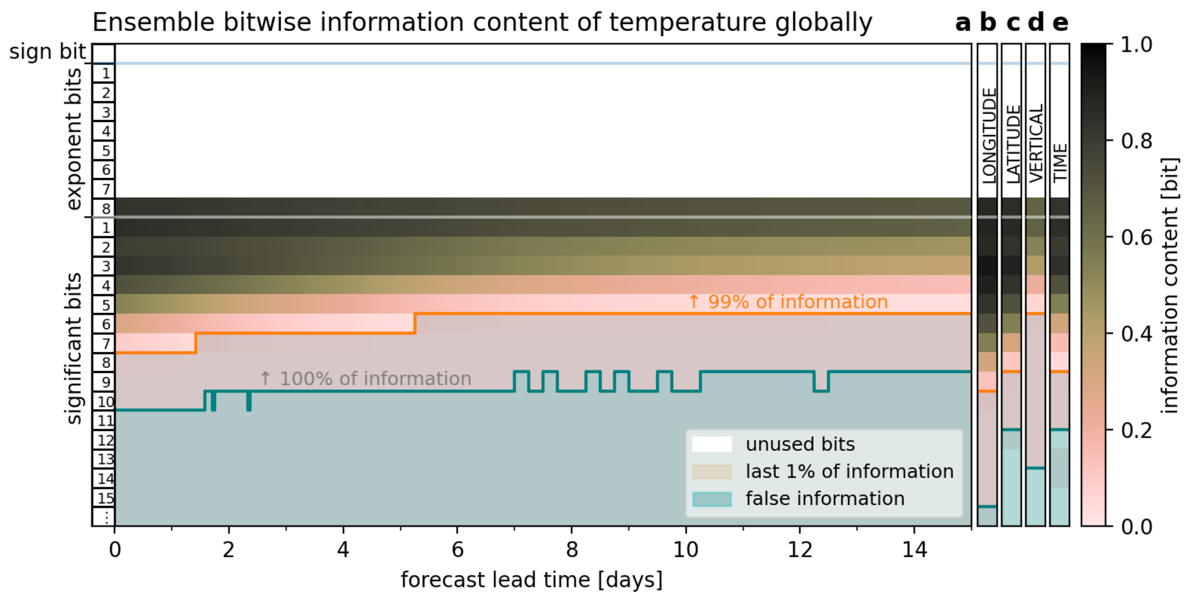

Supplementary Figure 10 | Bitwise real information content for temperature in various dimensions. a ensemble, **b** longitude, **c** latitude, **d** vertical and **e** forecast lead time. The ensemble information effectively encodes the ensemble mean, which is less information than in most other dimensions. Longitude, latitude and forecast lead time have the highest total information which should be preserved in compression. The ensemble information decreases over time as the ensemble spread increases.

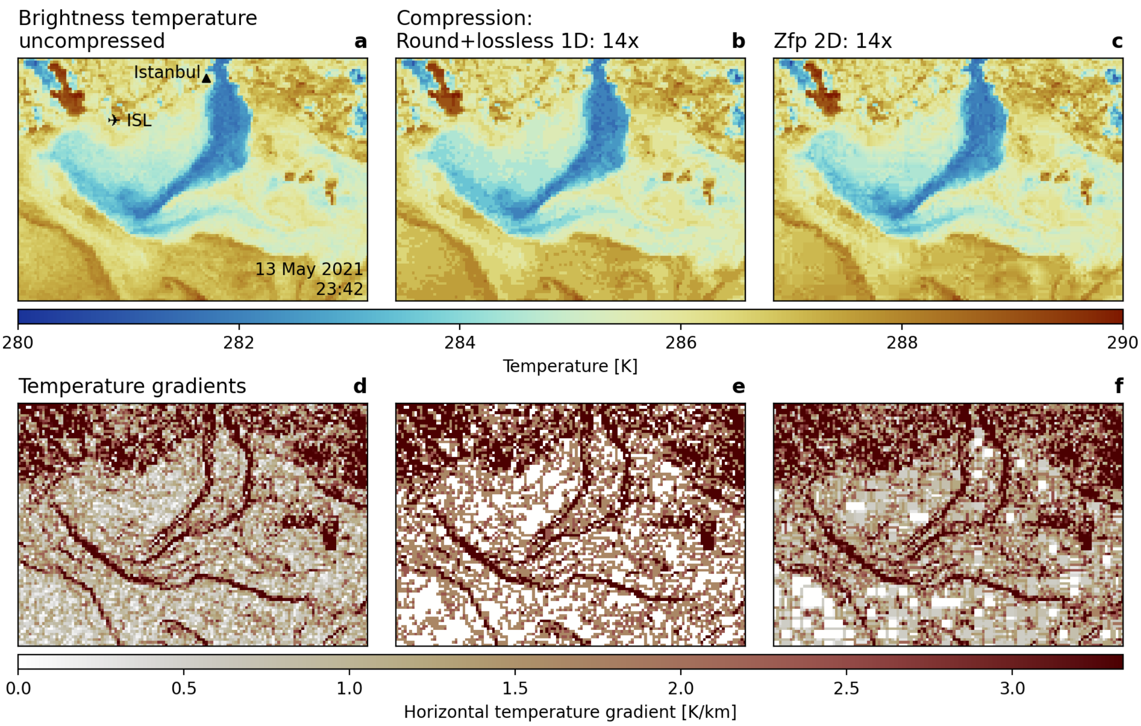

Supplementary Figure 11 | Preservation of gradients during compression. Compressing the brightness temperature of Fig. 7 (VIIRS sensor aboard the satellite Suomi NPP) south of Istanbul where the Black Sea outflows into the Marmara Sea. Oceanic fronts with strong horizontal gradients in sea surface temperature are visible. **a** Brightness temperature uncompressed. **b** as **a** but compressed using round+lossless preserving 99% of real information. **c** as **a** but using Zfp compression in the two horizontal dimensions. **d** Horizontal temperature gradient uncompressed highlighting the oceanic fronts from **a**. **e** as **a** but the horizontal gradient is calculated from the round+lossless compressed dataset as shown in **b**. **f** as **e** but using Zfp compression as shown in **c.** The coarseness of the visualisation represents the resolution of the data. Istanbul (Hagia Sophia) and Atatürk Airport (ISL) are marked for orientation.

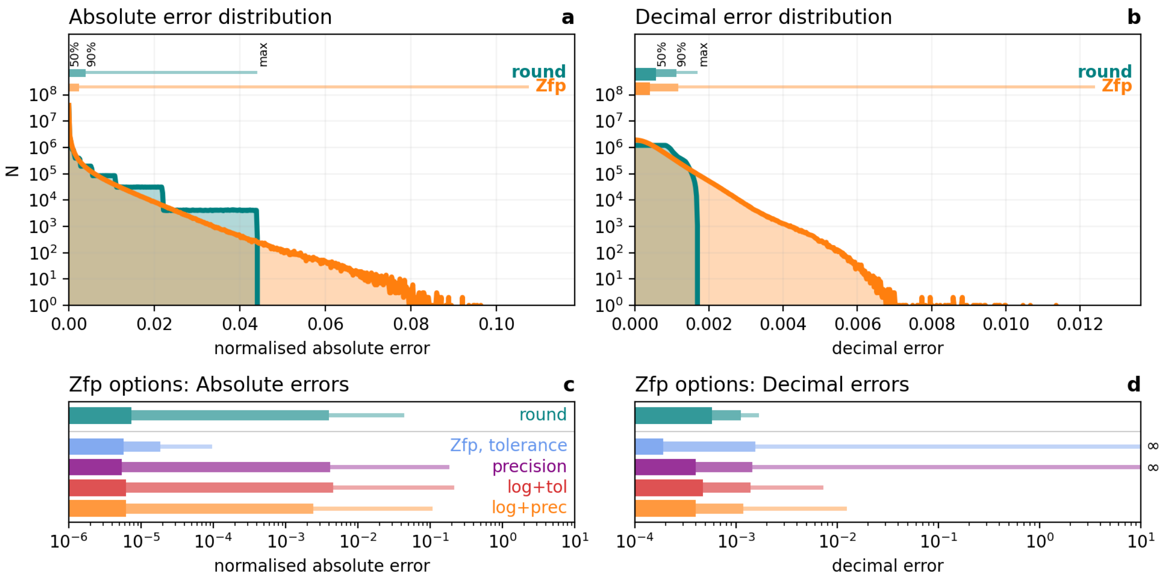

Supplementary Figure 12 | Error distribution of binary rounding compared to Zfp compression. IEEE round-to-nearest and Zfp compression of water vapour (specific humidity) in the three spatial dimensions. **a, c** normalised absolute errors **b, d** decimal errors. 7 mantissa bits are retained for rounding corresponding to 99% preserved information. The precision parameter of Zfp is chosen to yield median errors that are at least as small as those obtained by rounding. **c, d** Zfp via specifying tolerance (tol) or precision (prec) with and without log-preprocessing. Maximum decimal errors that reached infinity in **d** due to sign changes are marked.
